# Attachment of *Asaia bogorensis* Originating in Fruit-Flavored Water to Packaging Materials

**DOI:** 10.1155/2014/514190

**Published:** 2014-09-11

**Authors:** Dorota Kregiel, Anna Otlewska, Hubert Antolak

**Affiliations:** Institute of Fermentation Technology and Microbiology, Lodz University of Technology, 171/173 Wolczanska Street, 90 924 Lodz, Poland

## Abstract

The objective of this study was to investigate the adhesion of isolated spoilage bacteria to packaging materials used in the food industry. Microorganisms were isolated from commercial fruit-flavored mineral water in plastic bottles with flocks as a visual defect. The Gram-negative rods were identified using the molecular method through the amplification of a partial region of the 16S rRNA gene. Based on the sequence identity (99.6%) between the spoilage organism and a reference strain deposited in GenBank, the spoilage isolate was identified as *Asaia bgorensis*. Experiments on bacterial adhesion were conducted using plates made of glass and polystyrene (packaging materials commonly used in the beverage industry). Cell adhesion ability was determined using luminometry, plate count, and the microscopic method. The strain of *A. bogorensis* was characterized by strong adhesion properties which were dependent on the surface type, with the highest cell adhesion detected on polystyrene.

## 1. Introduction

Microbial spoilage of fruit-flavored mineral water usually originates during the production process. The raw materials, factory environment, the equipment, and packages, as well as lack of process hygiene, are possible sources of contamination [1]. Spoilage follows from metabolic processes that cause beverages to become undesirable or unacceptable for human consumption, due to changes to their sensory characteristics. Mineral waters with fruit juices or flavors are characterized by high water activity and high levels of vitamins and minerals, which make them good environments for the growth of spoilage microflora [2]. Due to the low pH level of these soft drinks, the predominant spoilage microfloras are acidophilic microorganisms which have developed tolerance towards preservatives used in beverage production. However, new exotic fruit ingredients used in soft drinks can introduce unusual spoilage species with unknown resistance to food preservatives.

The type of packaging used, such as cans and bottles, can also affect the development of spoilage microflora. The material may influence the number and type of cells that grow and adhere to the bottle surface, while the ability of microbial cells to adhere and accumulate on packaging materials can exacerbate contamination of the beverage, reducing its quality and microbiological safety [3, 4]. Packaging materials also vary greatly in terms of oxygen permeability. Glass is still the preferred packaging material for high quality fruit beverages, although the hot-fill/hold/cool process must be applied with care, in order to avoid container breakage. The growth of bacteria is also significantly enhanced by contact with the inner surface of bottles (the so-called bottle effect) [5]. Polystyrene (PS) is one of the plastic materials used most commonly in containers, lids, and bottles. PS is inexpensive, flexible, durable, and chemically resistant [6, 7]. However, the oxygen content in plastic bottles increases with time, whereas glass bottles are impermeable to oxygen [8].

The objective of this study was to identify the spoilage microflora that forms characteristic flocks in commercial bottled fruit-flavored mineral waters and investigate their bacterial adhesion to both glass and polystyrene packaging materials used in the food industry.

## 2. Materials and Methods

### 2.1. Isolation of Spoilage Microorganisms

Bacteriological analysis was performed on ten samples of spoiled commercial fruit-flavored mineral water (8.1% sucrose (w/v), 0.05% fruit flavor (w/v), 0.16% citric acid (w/v), 0.02% sodium benzoate (w/v), and 0.02% velcorin (w/v)) from polystyrene bottles. Quantitative examination of the samples was conducted using the pour plate method by inoculating GC agar medium (0.1 mL) with 2% D-glucose (w/v), 0.3% peptone (w/v), 0.3% yeast extract (w/v), and 0.7% CaCO_3_ (w/v) [9]. Incubation was conducted at 25°C. The characteristic colonies obtained were picked up from the plates, restreaked to ensure purity, and maintained at 20°C on GC agar slants.

### 2.2. Identification of Spoilage Bacteria

The following standard methods were used for identification: Gram staining, the aminopeptidase test (Bactident Aminopeptidase, Merck), the oxidase test (Bactident Oxidase, Merck), and the catalase test (Bactident Catalase, Merck). Identification was also performed using the PCR technique. For DNA extraction, the strain was cultured on Orange Serum Agar (Merck) for 24 h and the genomic DNA was isolated using a Genomic Mini Kit (A&A Biotechnology, Gdynia, Poland), according to the manufacturer's instructions.

The 16S rRNA gene was amplified by a polymerase chain reaction (PCR). The reaction was performed in a total volume of 50 *µ*L comprising 24 *µ*L REDTaq ReadyMix DNA polymerase (Sigma-Aldrich, St. Louis, MO, USA), 24 *µ*L PCR grade water, 1 *µ*L of template DNA (50 ng), and 0.4 *µ*L of each primer solution (100 *µ*M). A primer set with the sequences 5′-AGAGTTTGATCCTGGCTCAGAT-3′ and 5′-CGGCTACCTTGTTACGAC-3′ was used [10]. The reaction was carried out in a MJ Mini Thermal Cycler (Bio-Rad, Hercules, CA, USA) with initial denaturation at 94°C for 2 min, followed by 39 cycles of denaturation at 94°C for 1 min, primer annealing at 55°C for 1 min, elongation at 72°C for 3 min, and a final extension step at 72°C for 2 min.

The PCR products were separated using 1% agarose in 0.5 × TBE buffer with ethidium bromide and purified using a Clean Up Mini Kit (A&A Biotechnology, Gdynia, Poland), following the manufacturer's protocol. The nucleotide sequences of the detected bacterial strain were obtained using the BigDye Terminator Ready Reaction Cycle Sequencing Kit (Applied Biosystems, Foster City, CA, USA) and the reaction products were analyzed using an Applied Biosystems model 3730 Genetic Analyzer (Genomed, Warsaw, Poland). The nucleotide sequences then were compared with 16S rRNA gene sequences of* Asaia* sp. obtained from the National Center for Biotechnology Information (NCBI) using the program BLASTN 2.2.27+ (http://blast.ncbi.nlm.nih.gov/Blast.cgi) [11]. Multiple alignments of the sequences derived from the reference strain and the identified* Asaia* strains were performed using the Clustal W algorithm. Phylogenetic relationships were inferred using the neighbor-joining method in MEGA5 [12, 13]. No positions containing gaps were considered in the phylogeny analysis. All reconstructions were tested by bootstrapping with 1000 replicates. The evolutionary distances were computed using the maximum composite likelihood method and given in units of the number of base substitutions per site. The analysis involved 11 nucleotide sequences. The final dataset comprised a total of 1347 positions.

### 2.3. Bacterial Cultures

The isolated strain of* Asaia bogorensis* was stored in liquid GC medium (0.3% peptone (w/v), 0.3% yeast extract (w/v), and 0.7% CaCO_3_ (w/v)) with 2% of D-glucose (w/v) at 4°C [10]. Commercial flavored mineral water with saccharose as a carbon source was used for aerobic cultivation. Due to the presence of thermosensitive ingredients, this medium was sterilized by filtration using a 0.45 *µ*m pore-size Millipore filter. The culture medium (20 mL) was then poured into 25 mL Erlenmeyer flasks, into which sterile carriers were placed vertically in such a way that half of the carrier was immersed in the medium, while the other part remained outside. The amount of inoculum was standardized to obtain a cell concentration in the culture medium approximately equal to 5000–10000 CFU/mL at the start of the experiment. The samples were incubated at 25°C on a laboratory shaker (135 rpm) for 10 days.

### 2.4. Solid Carriers

Two main types of material were assessed: Star Frost 76 × 26 mm white glass slides (G) (Knittel Glass, Germany), used as the reference material, and rectangular discs of polystyrene (PS) 76 × 26 mm (Paccor Packaging Poland, Skierniewice), a material certified by the Polish National Institute of Public Health and approved for contact with food.

### 2.5. Determination of Contact Angle and Surface Tension

The contact angle values for the two different solvents, dimethylformamide (DMF) and water, were determined using a ramé-hart NRL goniometer (ramé-hart instrument co., Succasunna, NJ, USA) equipped with a JVC KYF-70B camera (JVC, Yokohama, Japan). The dynamic contact angle was calculated using DROPimage software (ramé-hart instrument co., Succasunna, NJ, USA), as the average of 15 measurements. The total surface tension was calculated from the values of the contact angles of solvents with different polarities (Owens-Wendt method) [10].

### 2.6. Assessment of Bacterial Adhesion

The analysis of adhesion to the carriers was performed using luminometry, the plate count method, and microscopic observations. For the luminometric tests, the carrier plate was removed from the culture medium, rinsed with sterile distilled water and swabbed using free ATP sampling pens (Merck, Germany). Measurements were reported in relative light units (RLU) using a HY-LiTE 2 luminometer (Merck) [10]. The colony count method was used to determine the number of viable bacterial cells, both on the tested surface and in the culture medium. The carrier plate was removed from the culture medium, rinsed with sterile distilled water, and swabbed using sterile swabs for surface testing. The bacterial suspensions were vortexed vigorously in 0.85% saline solution with 0.1% Tween 80 for 5 min and transferred onto GC agar. After incubation (25°C, 96 h), the characteristic pink colonies of* Asaia* spp. were counted and the number of attached bacterial cells per square centimetre of the carrier was determined. The relative adhesion coefficient (*A*) was then calculated: the total number of adhered cells was divided by the total number of planktonic cells in the given sample. The coefficient* A* was expressed as a percentage value [14].

In the microscopic studies,* Asaia* spp. cells were stained with basic fuchsin (0.5%). Bacterial cells on the carrier were observed using an OLYMPUS type BX41 light with DP72 digital camera. The total cell adhesion area in the observation field was evaluated using UTHSCA Image Tool software (http://compdent.uthscsa.edu/dig/itdesc.html).

Mean values were calculated from the data obtained from the three independent experiments. Comparisons between the mean values were performed using the one-way ANOVA test (STATISTICA 10, StatSoft, Poland).

## 3. Results and Discussion

### 3.1. Isolation and Identification of Spoilage Microorganisms

**Figure 1:**
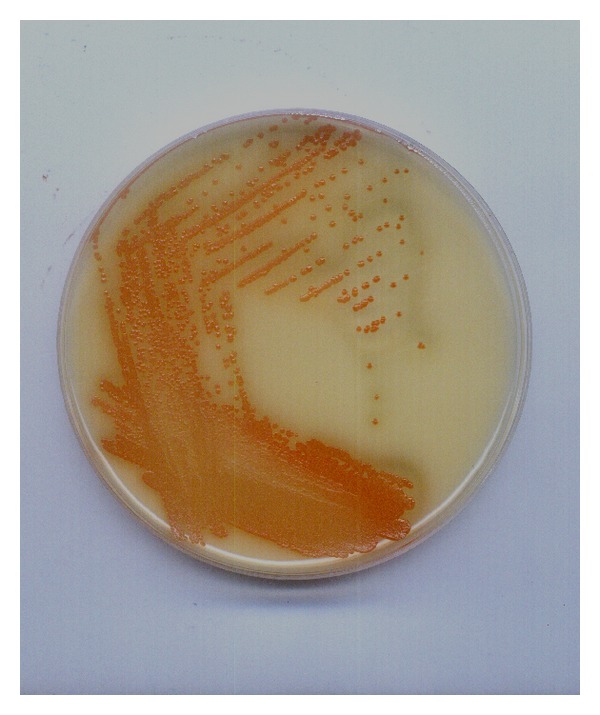
Growth of the dominant morphotype of AAB on GC agar medium.

The samples of spoiled fruit-flavored mineral water (pH = 3.3) had characteristic, visually observable flocks. Incubations demonstrated the presence of mixed cultures consisting of two main morphotypes, from which single colonies were isolated. One of these morphotypes was the yeast* Rhodotorula* spp. However, the second was dominant and very characteristic—the bacteria grew best at 25°C on agar medium with glucose, peptone, and CaCO_3_, forming small, pale, smooth, orange-pink colonies with clear zones after 72 h of incubation (Figure 1). Cells of this morphotype were Gram negative, catalase positive, oxidase negative, and rod-shaped, measuring 0.5–1.0 × 0.8–2.0 mm. The phenotypic characteristic of the isolate obtained from the beverage was consistent with that described previously for acetic acid bacteria of the genus* Asaia*.

**Figure 2:**
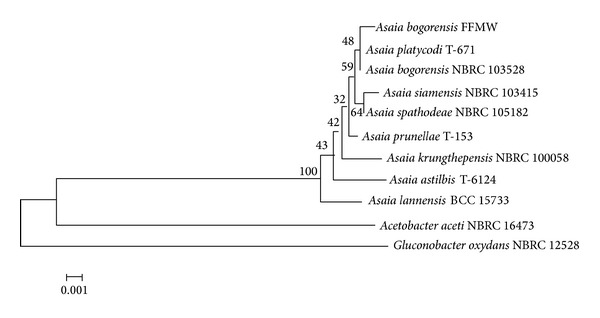
The phylogenetic tree constructed on the basis of 16S rRNA gene sequences of* Asaia* reference strains,* Gluconobacter oxydans* and* Acetobacter aceti*. The tree was constructed using the neighbor-joining method and tested by bootstrapping (1000 replicates). Only branches with 50% and above support from bootstrapping were shown.

AAB are known for the high frequency of their spontaneous mutations [15]. Therefore, polyphasic procedures should be followed based on both classical phenotypic tests and genotypic methods. It has been shown that using a single feature as the sole diagnostic criterion for AAB species definition is unreliable. In our study, API identification tests were also unable to identify the AAB with high confidence (unacceptable profiles), so identification was additionally based on the 16S rRNA gene sequence. Direct sequencing of the 16S rRNA gene identified the cells as* A. bogorensis* (99.6% identity with* Asaia bogorensis* NBRC 103528). The phylogenetic relationships determined for the 16S rRNA gene sequences of 8 strains belonging to* Asaia* spp. (*A. bogorensis*,* A. siamensis*,* A. krungthepensis*,* A. lannensis*,* A. spathodea*,* A. astilbis*,* A. platycodi*, and* A. prunellae*) and 2 strains of* Gluconobacter oxydans* and* Acetobacter aceti* are shown in Figure 2. The 16S rRNA gene sequence determined for* Asaia bogorensis* strain FFMW was deposited in the GenBank database with accession number KC756841.

*Asaia bogorensis* strains were first isolated from orchid tree flowers (*Bauhinia purpurea*) and plumbago flowers (*Plumbago auriculata*), as well as from fermented glutinous rice by Yamada et al. [16]. The hydrophilic cells are able to synthesize extracellular cellulose in a solid pellicle at the air-liquid interface of static culture media [17, 18]. This ability may contribute to the formation of biofilms on many types of surface commonly used in food processing [4]. The results obtained from our experiments suggest that* Asaia bogorensis* is able to grow in fruit-flavored mineral water despite the combination of low pH and chemical preservatives (sodium benzoate, citric acid, and dimethyl dicarbonate, velcorin). This property has also been noted in studies conducted by Horsáková et al. [3].* A. bogorensis* does not appear to present a significant risk to human health, although some strains may be opportunistic pathogens in patients with reduced immunity [19–21].

### 3.2. Surface Tension of Packaging Materials

Surface tension is one of the most important physicochemical properties of any solid surface and is correlated with resistance to biofilm formation. A general relationship between surface tension and the relative amount of adhesion was found to form a “Baier curve,” with the zone of minimal adhesion in the region between 20 and 30 mJ/m^2^. High bioadhesion occurred when this parameter exceeded 30 mJ/m^2^ [22]. The values for tested glass and polystyrene were different (their measured surface energies varied from 40 to 54 mJ/m^2^) but in both cases they exceeded the critical value of 30 mJ/m^2^. The polystyrene exhibited lower surface tension than the glass surface (Table 1). This fact might indicate that PS is more adhesive. However, the adhesion properties of tested surfaces can become modified rapidly by immersion in water and by the adsorption of conditioning films [23]. In our study, the tested surfaces were in contact with fruit-flavored mineral water containing organic matter for 10 days, which may have influenced the adhesive events associated with the attachment of bacterial cells.

### 3.3. Attachment of* Asaia bogorensis* to Packaging Materials

**Figure 3:**
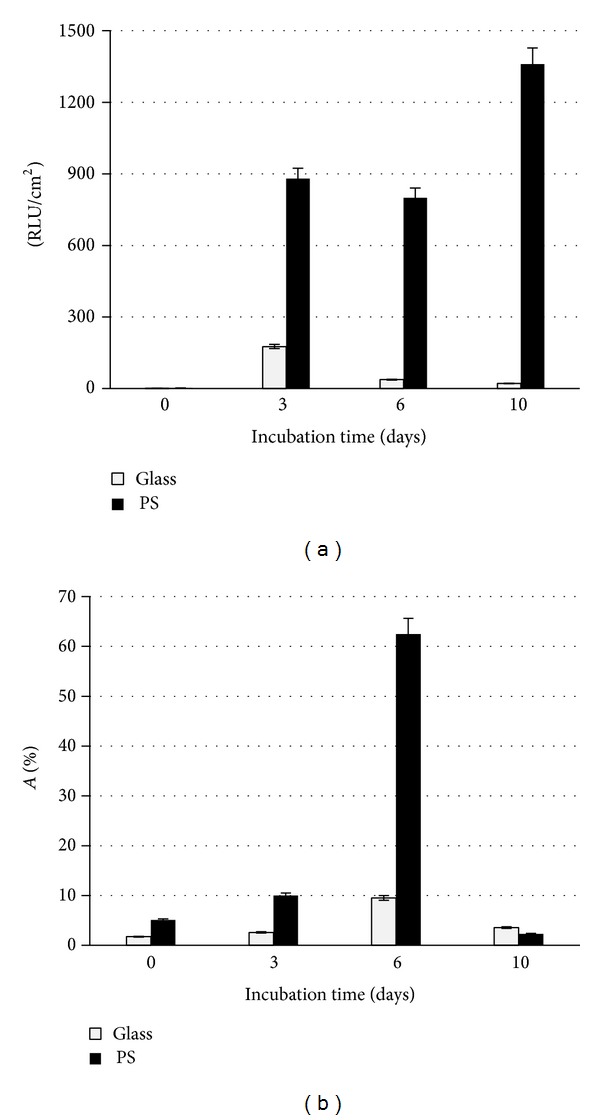
Adhesion of* A. borogensis* in fruit-flavored mineral water: (a) adhesion reported in relative light units per cm^2^ and (b) relative adhesion coefficient (*A*) expressed in %.

Biofilm formation may be a survival strategy for starved bacteria, and it has been confirmed that increased cell adhesion is often correlated with nutrient limitation [23]. Therefore, our adhesion studies used commercial flavored mineral water that was poor in nutrients. The intensity of biofilm formation on two packaging materials, glass and polystyrene, was assessed by luminometric measurement and expressed in relative light units (RLU).  Figure 3 presents the results of luminescence (RLU/cm^2^) and the relative adhesion coefficient* A* (%) for all tested surfaces. Biofouling during incubation had a dynamic and changeable character. The number of attached* A. bogorensis* cells was significantly higher with polystyrene. After 10-day incubation, the levels of adhesion to glass and PS in the flavoured water were 22 and 1360 RLU/cm^2^, respectively. The relative adhesion coefficient for polystyrene was also several times higher in comparison to the glass surface. Similar results had been obtained by Kregiel [24] with the* Asaia lannensis* strain and polyethylene material.

**Figure 4:**
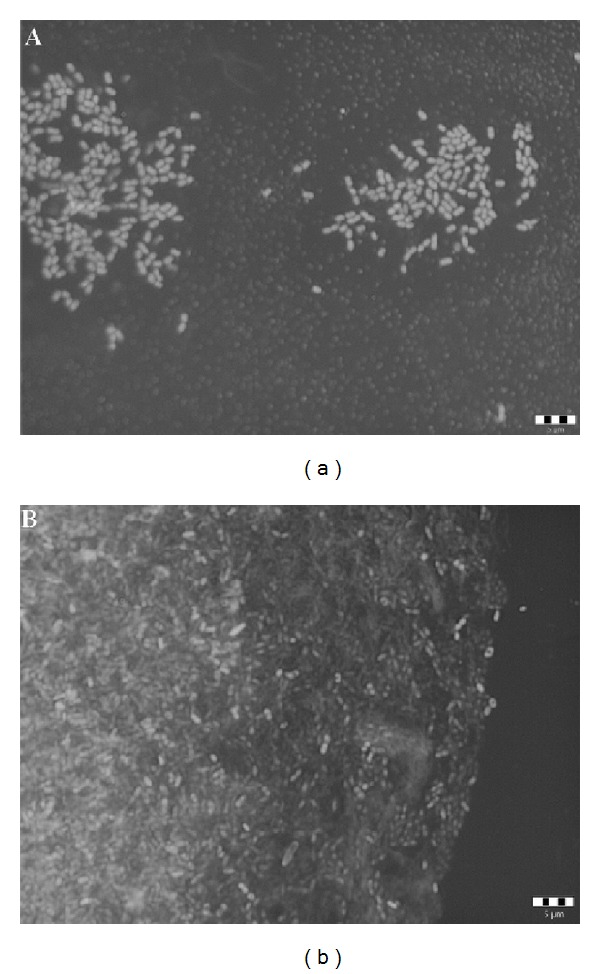
Attachment of* A. bogorensis* cells to (a) glass and (b) polystyrene. Bars represent 5 *μ*m.

In the experiments we also used qualitative analysis of adherent cells based on light microscopy.  Figure 4 shows images of glass and polystyrene surfaces stained with fuchsin. Irregular cell adhesion with an extracellular substance was detected on the PS material, resulting in surface coverage ranging from approximately 30% to 50% of the total area. Therefore, glass proved to be more resistant as a packaging material to* Asaia bogorensis* adhesion.

## 4. Conclusion

This study has identified the presence of* Asaia bogorensis* in samples of defective strawberry-flavored bottled mineral water. The low pH level and addition of chemical preservatives in these products did not prevent the growth of bacterial cells. Adhesion and biofilm formation on the bottle materials were shown to exacerbate contamination by* A. bogorensis*, reducing the quality and microbiological safety of the beverage products. Attachment of* A. bogorensis* cells decreased as the polar surface tension of the substrate increased. Using glass as packaging material, with a high polar contribution of surface tension, allows for a significant reduction of* Asaia* spp. adhesion and may contribute to improving the microbiological stability of fruit-flavored mineral waters.

## Figures and Tables

**Table 1:** The surface free energy (SFE) of solid surfaces [mJ/m^2^].

Surface	Total SFE	Dispersive contribution of SFE	Polar contribution of SFE
Glass	54.2 ± 0.4	7.9 ± 0.1	44.3 ± 0.3
Polystyrene	40.6 ± 0.3	34.3 ± 0.2	6.3 ± 0.1
